# Prevalence and Correlates of Anti-DSG2 Antibodies in Arrhythmogenic Right Ventricular Cardiomyopathy and Myocarditis: Immunological Insights from a Multicenter Study

**DOI:** 10.3390/jcm13226736

**Published:** 2024-11-08

**Authors:** Andrea Silvio Giordani, Elena Pontara, Cristina Vicenzetto, Anna Baritussio, Maria Grazia Peloso Cattini, Elisa Bison, Federica Re, Renzo Marcolongo, Shaylyn Joseph, Diptendu Chatterjee, Meena Fatah, Robert M. Hamilton, Alida Linda Patrizia Caforio

**Affiliations:** 1 Cardiology, Department of Cardiac Thoracic Vascular Sciences and Public Health, University of Padova, Via Giustiniani 2, 35128 Padova, Italy; 2 Cardiology Division, San Camillo Hospital, 00152 Rome, Italy; 3 Department of Pediatrics, The Labatt Family Heart Centre and Translational Medicine, The Hospital for Sick Children & Research Institute, The University of Toronto, Room 1725D, 555 University Avenue, Toronto, ON M5G 1X8, Canada

**Keywords:** myocarditis, ARVC, arrhythmogenic cardiomyopathy, Desmoglein-2, antibodies, ELISA, anti-heart antibodies, anti-intercalated disk antibodies

## Abstract

**Background:** Autoantibodies against Desmoglein-2 desmosomal protein (anti-DSG2-ab) were identified in Arrhythmogenic Right Ventricular Cardiomyopathy (ARVC) by Enzyme-Linked ImmunoSorbent Assay (ELISA); anti-intercalated disk autoantibodies (AIDAs) were identified in myocarditis and (ARVC) by indirect immunofluorescence (IFL). We aim to assess: (1) anti-DSG2-ab specificity in ARVC and myocarditis, (2) accuracy of anti-DSG2-ab detection by ELISA versus AIDA by IFL, and (3) clinical correlates of anti-DSG2-ab in ARVC. **Methods:** We included 77 patients with ARVC, 91 with myocarditis/dilated cardiomyopathy (DCM), 27 with systemic immune-mediated diseases, and 50 controls. Anti-heart antibodies (AHAs) and AIDAs were assessed by IFL, and anti-DSG2-ab by ELISA (assessed both by optical density, OD, and U/L). Receiving operator curve (ROC) analysis was used to assess ELISA diagnostic accuracy. **Results:** A relevant proportion (56%) of ARVC patients was anti-DSG2-ab-positive, with higher anti-DSG2-ab levels than controls. Anti-DSG2-ab titer was not different between ARVC and myocarditis/DCM patients (48% anti-DSG-ab positive). Frequency of anti-DSG2 positivity by ELISA was higher in AIDA-positive cases by IFL than AIDA-negative cases (*p* = 0.039 for OD, *p* = 0.023 for U/L). In ARVC, AIDA-positive patients were more likely to be AHA-positive (*p* < 0.001), had pre-syncope (*p* = 0.025), and abnormalities in cardiac rhythm (*p* = 0.03) than ARVC AIDA-negative patients, while anti-DSG2-ab positivity did not have clinical correlates. **Conclusions:** Anti-DG2-ab detection in ARVC and myocarditis/DCM reflects immune-mediated pathogenesis to desmosomal proteins. Higher frequency of anti-DSG2-ab positivity by ELISA by U/L was higher in AIDA-positive cases by IFL than AIDA-negative cases, in keeping with the hypothesis that DSG2 is one of AIDA autoantigens. In ARVC, AIDA status but not anti-DSG2-ab showed distinct clinical correlates, possibly reflecting a wider AIDA autoantigenic spectrum.

## 1. Introduction

Arrhythmogenic Right Ventricular Cardiomyopathy (ARVC) is a rare cardiac disease characterized by distinct electrocardiographic, morphological, and histological alterations, in particular fibro-fatty replacement of the myocardium [1]. ARVC (OMIM identifier: 609040) has a heredo-familial pattern, and patients are at increased risk of sudden cardiac death, even at an early age, due to sustained ventricular arrhythmias. In fact, ARVC is recognized as one of the major causes of cardiac death in the young [2]. Right ventricular involvement can be present in isolation as in the “classical” ARVC form but, as a recent consensus document pointed out, isolated left ventricular (“left dominant”) or biventricular involvement are part of the spectrum of the disease [3]. Arrhythmogenic cardiomyopathy (AC) diagnosis can be achieved through a multiparametric approach, recently detailed in the so-called “Padua criteria” [4].

The identification of a pathogenic or likely pathogenetic genetic mutation linked to ARVC/AC is one of the cornerstones for diagnosis. In the ARVC classical form, mutations involve genes encoding desmosome proteins, such as Desmoglein 2 (DSG2) [5], Plakophilin 2 (PKP2), Plakoglobin (JUP) [6], Desmoplakin (DSP) [7], and Desmocollin 2 (DSC2) [8]. Desmosomal proteins form the intercalated disks, which are specialized intercellular junctions between cardiomyocytes [9]. Remarkably, autoantibodies against desmosomal proteins, i.e., anti-intercalated disk autoantibodies (AIDAs) have been identified by IFL both in myocarditis [10] and in ARVC patients [11], as previously described [10,12]. AIDA antigen specificity has not yet been defined, since desmosomes are complex structures composed of a variety of different proteins. It is ascertained that mutations in certain desmosome proteins can lead to loss of intercellular adhesions, cell death, myocardial fibro-fatty replacement, and altered electrical signaling, leading to both cardiac and extra-cardiac abnormalities [13]. The mechanism leading to desmosome destruction may be immune mediated, but this hypothesis has not been fully elucidated. Thus, the exact pathogenesis of ARVC has not been fully elucidated. A growing amount of evidence suggests that inflammatory processes take part in the disease progression: in a sizable proportion of ARVC patients, the clinical course is characterized by the so called “hot phases” with infarct-like chest pain, troponin release, and evidence of inflammation on noninvasive imaging, especially cardiac magnetic resonance (CMR) [14]. In certain cases, endomyocardial biopsy (EMB) or autopsy findings in ARVC have revealed autoimmune myocarditis, characterized by inflammatory infiltrates without detectable viral agents [15]. Moreover, a recent monocentric ARVC study revealed the presence of high titer autoimmune markers traditionally associated with myocarditis, i.e., organ-specific anti-heart autoantibodies (AHAs) and AIDAs in most (85%) ARVC patients and in a relevant proportion (45%) of their relatives [16]. In biopsy-proven myocarditis and systemic disorders with myocardial involvement, AHA and AIDA positivity have a proven diagnostic and prognostic value [10,17,18,19]. In 2018, Chatterjee et al. [11] used Western Blot to prove that anti-desmosome antibodies are present in ARVC patients and in an animal model of the disease, with both diagnostic and prognostic implications. They postulated that this antibody identified a disease-specific DSG2 epitope, contributing to the pathogenesis of the disease through loss of tolerance or release of immunogenic “cryptic” epitopes after desmosome disruption. They found that anti-DSG2 autoantibodies (anti-DSG2-abs) were specific for ARVC and absent in healthy subjects and patients with other forms of heritable cardiomyopathy, but myocarditis patients were not included in the study.

Finally, from a technical perspective, anti-DSG2-abs were tested by Chatterjee et al. with the Enzyme-Linked ImmunoSorbent Assay (ELISA) method, while the only validated technique for AHA and AIDA assessment is immunofluorescence (IFL) [10], and a correlation between the two methods in ARVC has not been explored so far.

The aims of our study are to: (1) assess the disease specificity of anti-DSG2-ab in ARVC, myocarditis, and other extracardiac immune-mediated diseases, (2) assess the correlation of AIDA-positive status by IFL and anti-DSG2-ab detection by ELISA, and (3) to assess the clinical and/or prognostic correlates of anti-DSG2-abs in ARVC.

## 2. Methods

### 2.1. Study Design and Data Collection

This study retrospectively included four different groups of patients:

(1) ARVC patients: these patients were followed up at the Cardiomyopathy Unit of San Camillo Hospital in Rome, Italy. ARVC diagnosis was based on the 2010 revised diagnostic Task Force criteria [20]: all patients underwent 12-lead ECG, signal-averaged ECG, 24 h Holter monitoring, transthoracic Doppler echocardiography, and CMR; cardiac catheterization and EMB were performed at the clinicians’ discretion. Genetic testing was also performed when clinically indicated. Demographic and clinical data have been collected.
(2) Myocarditis and dilated cardiomyopathy (DCM) patients: these patients were followed-up either at the Cardiomyopathy Unit of San Camillo Hospital in Rome, Italy, or at the Cardiological Department of Padua University Hospital in Padova, Italy. Diagnosis was based on ECG, echocardiographic, and CMR findings; EMB was performed when clinically indicated.
(3) Systemic immune-mediated disease (SID) or autoimmune disease patients: sera from patients with extra-cardiac SIDs or other extra-cardiac autoimmune conditions from Padua University Hospital in Padova, Italy, were tested.
(4) Controls: this group includes sera obtained from healthy volunteers, i.e., free from cardiac and non-cardiac diseases, and from patients with non-inflammatory cardiac or extra-cardiac diseases. Controls were recruited at the Padua University Hospital in Padova. In addition, 10 control sera (commercial sources) were tested.

This study’s protocol followed the ethical guidelines of the Declaration of Helsinki; Institutional Review Board approval was obtained at San Camillo Hospital in Rome and Azienda-Ospedale Università di Padova Hospital (protocol number 0027841).

### 2.2. Antibody Detection Techniques

Anti-DSG2-abs were measured through ELISA. Three chemically synthesized peptides of human DSG-2 protein [11,21] were diluted in antigen coating buffer (Immunochemistry technique) and coated at the final concentration of 2.5 µg/mL to a microtiter plate (Corning Costar 96-well EIA Plate) and incubated at 4 °C overnight. After three washes with ELISA wash buffer, the wells were incubated with a blocking solution (Tris buffer saline solution, TBS, containing a 0.5% protease free bovine serum albumin, BSA, Sigma) for 2 h at room temperature (RT). After four washes, the plate was incubated with serum samples diluted 1:100 in the Neptune sample diluent (Immunochemistry technologies) at RT for two hours. A reference curve was made with scaling dilutions of positive control’s serum. After three washes, the wells were incubated with an HRP Affipure Goat anti-human IgG conjugated with peroxidase diluted 1:20,000 in HRP Conjugate Stock Stabilizer (Immunochemistry technologies) at RT for two hours. After three washes, a peroxidase substrate solution (TMB) was added and kept in the dark for two minutes. Then, the reaction was stopped with a 2 M solution of Sulfuric Acid. The optical density (OD) was determined using an ELISA plate reader at 450 nm. The anti-DSG2-ab values were also expressed in U/dL, taking as 100 the first dilution of positive control (patient with the highest level measured) used as a reference curve.

With respect to AHA and AIDA, their assessment was performed by standardized IFL, as previously described [10,13,14,15,16,18]. In brief, patients’ sera were tested by indirect IFL at a dilution of 1/10 on 4 μm-thick cryostat sections of blood group 0 normal human atrium and skeletal muscle. Two sera were used as standard positive and negative controls and titrated in every assay. All sera were read blindly against these standards using a fluorescence microscope (Zeiss Axioplan 2 imaging, Zeiss, New York, NY, USA). An additional positive control serum was titrated to assess reproducibility. End point titers for this serum were reproducible within one double dilution in all assays.

### 2.3. Statistical Analysis

Categorical variables are reported as absolute values and percentages (relative frequencies), and continuous variables are reported using median and interquartile range (IQR, 1st–3rd quartile). Continuous variables between patient groups were compared using the T-Student test for normally distributed variables, or the Kruskal–Wallis or Mann–Whitney tests for non-normally distributed variables; normality of variable distribution was verified using the Shapiro–Wilk or Kolmogorov–Smirnov test. Categorical variables were compared using the Chi-squared test or the Fisher exact test. For all tests, a two-sided *p*-value < 0.05 was considered significant. The Bonferroni correction was applied to adjust *p*-values for multiple comparisons when appropriate (p_adj_). Receiving operator curve (ROC) analysis was used to assess ELISA diagnostic accuracy [22]. Data were collected using Microsoft Excel data sheets, and statistical analysis was performed using the SPSS software version 28.0.1.0.

## 3. Results

### 3.1. ARVC Patients’ Clinical and Immunological Characteristics

Seventy-seven patients with ARVC were included, with a median age of 44, 50 years (IQR 27.75–54.00); 56% were male (Table 1). The majority (71%) of patients was in New York Heart Association (NYHA) functional class I at diagnosis, with only a minority showing severe heart failure symptoms (4% in class III-IV). At diagnosis, the most commonly reported symptoms were pre-syncope (38%), syncope (11%), or palpitations (11%). At diagnosis, biventricular function was overall preserved; median left ventricular ejection fraction (LVEF) was 62% (IQR 56–66) and median right ventricular area fractional shortening (RV AFS) was 48.5% (IQR 38–56). In a sizable proportion of patients, ventricular arrhythmias were present: 17% patients showed non-sustained ventricular tachycardia (NSVT) and 2% sustained VTs. At follow-up, 40% of patients had ongoing cardiac symptoms, and in 11%, the implantation of a permanent cardiac defibrillator (ICD) was indicated. At the ECG Holter monitoring, the median number of ventricular ectopic beats was 641 (IQR 8–10,224). Genetic analysis showed pathogenic or likely-pathogenic mutations in ARVC-related genes in 15 patients (19%): in 3 cases, a pathogenic variant of the Desmoglein gene was identified, in 3 cases of Desmoplakin, and in 9 cases of Plakophilin (Supplementary Table S1); in the remaining patients, genetic testing was either negative or non-available. With respect to immunological biomarkers, a relevant proportion of ARVC patients (37%) showed AHA positivity, and 7% were AHA strong-positive. Moreover, 10% were AIDA-positive, and in 9% of cases, ARVC patients were positive for both AHA and AIDA. With respect to anti-DSG2 antibodies, median anti-DSG2 antibody levels were 5.39 U (IQR 3.07–9.175) and 277.05 OD (IQR 170.75–423.38).

### 3.2. Overall Cohort Anti-DSG2-ab Evaluation

The overall cohort (N = 245) included four groups of patients: 77 patients (31%) had ARVC diagnosis, 90 (37%) had myocarditis or non-ischemic DCM, 50 (20%) had an extracardiac autoimmune or immune-mediated disease, and 27 (11%) were healthy controls (Table 2). The median age was 43 years (IQR 28–54); nearly half of the whole cohort (49%) were male. In the overall cohort, 40% of individuals were AHA-positive, and in particular, 8% were AHA strong-positive; 26% were AIDA-positive, and in particular, 2% AIDA strong-positive. In 20% of cases, AHA and AIDA were both positive.

**Figure 1:**
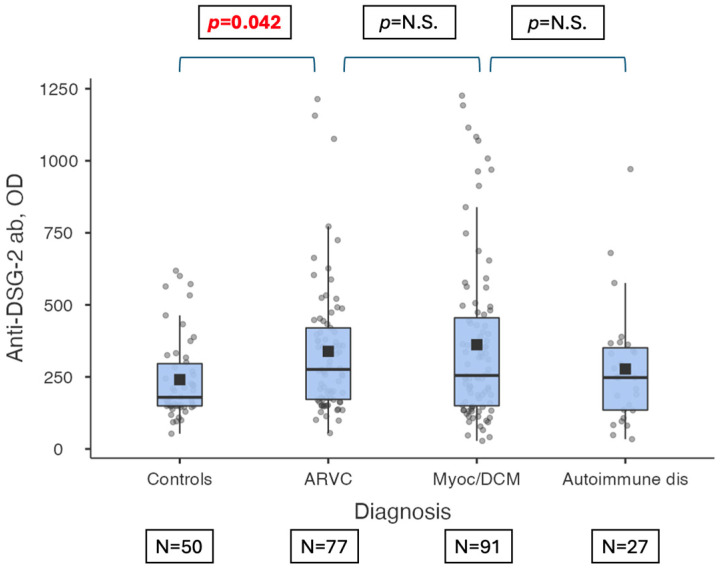
Anti-DSG-2-ab levels according to patients’ diagnostic group (i.e., healthy controls, ARVC, myocarditis/DCM, and autoimmune extracardiac diseases), evaluated as optical density (OD). The horizontal lines indicate the result of comparison of anti-DSG2-ab levels according to diagnostic group; anti-DSG2-ab levels were significantly higher in ARVC patients than healthy controls (*p* = 0.042). Legend: Anti-DSG2-ab: anti-Desmoglein-2 antibody, ARVC: arrhythmogenic right ventricular cardiomyopathy, Autoimmune dis: autoimmune extracardiac diseases, Myoc/DCM: Myocarditis/Dilated Cardiomyopathy, N.S.: non-significant, OD: optical density, *p*: *p*-value.

**Figure 2:**
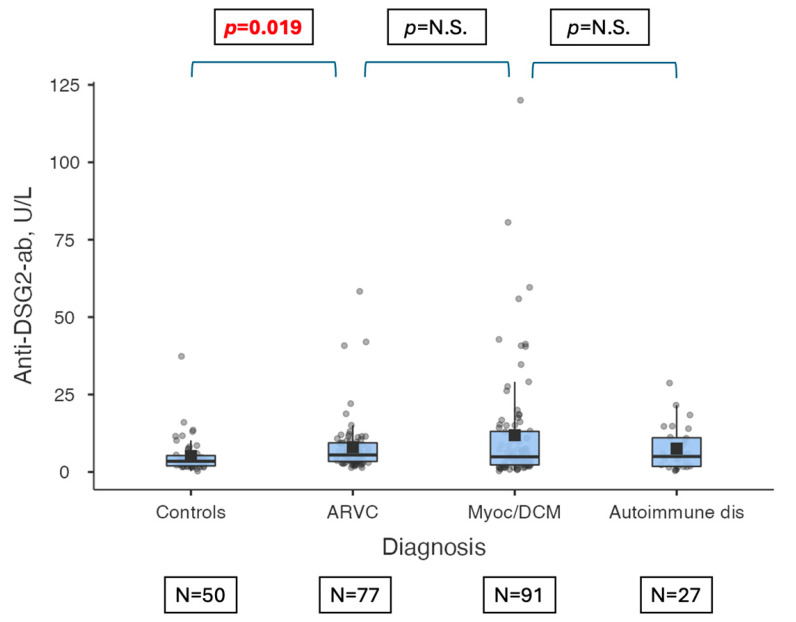
Anti-DSG-2-ab levels according to patients’ diagnostic group (i.e., healthy controls, ARVC, myocarditis/DCM, and autoimmune extracardiac diseases), evaluated as U/L. The horizontal lines indicate the result of comparison of anti-DSG2-ab levels according to diagnostic group; anti-DSG2-ab levels were significantly higher in ARVC patients than healthy controls (*p* = 0.019). Legend: Anti-DSG2 ab: anti-Desmoglein-2 antibody, ARVC: arrhythmogenic right ventricular cardiomyopathy, Autoimmune dis: autoimmune extracardiac diseases, Myoc/DCM: Myocarditis/Dilated Cardiomyopathy, N.S.: non-significant, *p*: *p*-value.

Median levels of anti-DSG2 antibodies were compared between the four groups, according to both OD (Table 3, Figure 1) and U/L concentration assessment (Table 4, Figure 2). Considering both methods, ARVC patients had higher levels of anti-DSG2 antibodies than controls (p_adj_ = 0.042 by OD, p_adj_ = 0.019 by U/L). Moreover, according to both methods, anti-DSG2 antibody titer was not different between ARVC (276 by OD, IQR 172–420; 5.5 by U/L, IQR 3.4–9.4, Table 2) and myocarditis/DCM patients (255 by OD; IQR 150–455; 4.9 by U/L, IQR 2.28–13.1, Table 2; p_adj_ not significant for both, Table 3 and Table 4).

### 3.3. ELISA and IFL Correlation

**Figure 3:**
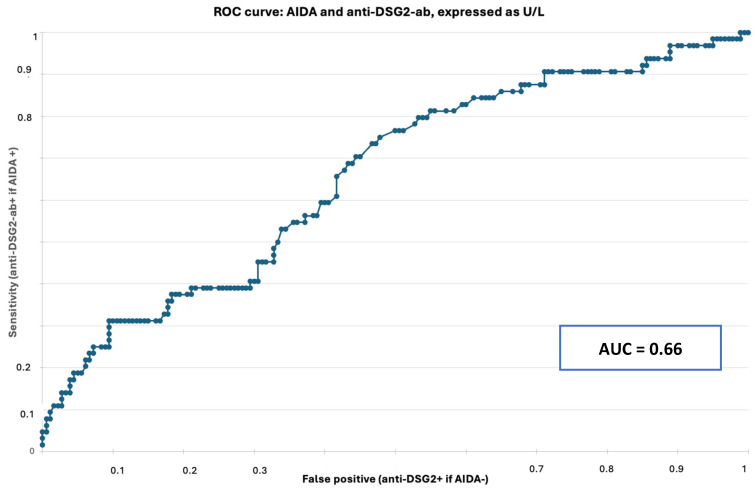
Standard ROC (Receiving Operator Curve) curve for anti-DSG2-antibodies, expressed as U/L, in the whole study cohort (N = 245). The Area Under the Curve (AUC) value indicates a sufficient diagnostic accuracy of anti-DSG2-ab detection by Enzyme-Linked ImmunoSorbent Assay (ELISA) with respect to anti-intercalated disk autoantibodies (AIDAs) by indirect immunofluorescence (IFL). Legend: AIDA: anti-intercalated disk antibody, AUC = area under the curve, anti-DSG2 ab: anti-Desmoglein-2 antibody.

The Receiving Operator Curve (ROC) method was used to assess ELISA result accuracy compared with the reference method, i.e., AIDA status tested by IFL. The accuracy of anti-DSG2-ab detection by ELISA was similar when assesses by OD (AUC = 0.6053), Supplementary Figure S1) and when assessed by U/dL (AUC = 0.66, Figure 3). Considering the higher technical reproducibility of the assessment by U/dL rather than OD, we used anti-DSG2-ab U/L as reference values (+2 standard deviations) for classifying patients as anti-DSG2-ab-positive (cut-off 5.12 U/L): ARVC patients were anti-DSG2-ab-positive in 51% of cases (39/77), and myocarditis/DCM patients in 48% of cases (44/91).

To further verify if AIDA positivity, assessed by IFL, and anti-DSG2-ab positivity, assessed by ELISA, were correlated, we compared the proportion of AIDA- and anti-DSG2-positive patients in the overall cohort. In the whole study population (N = 245), we observed higher frequency of anti-DSG2 positivity by ELISA in AIDA-positive cases by IFL than AIDA-negative cases (*p* = 0.039) (Supplementary Table S2). Similarly, we observed a higher frequency of AHA positivity in anti-DSG2-positive cases (*p* = 0.002) when assessed by U/dL (Supplementary Table S3).

### 3.4. Clinical and/or Prognostic Immunological Correlates of Immunological Markers in ARVC

ARVC patients (N = 70) were classified as anti-DSG2-ab-positive (N = 38, 54%) and negative (N = 32, 46%) according to the 2 SD U/dL cut-off (Supplementary Table S4). No significant differences were observed between ARVC anti-DSG2-ab-positive and negative patients with respect to any of the analyzed clinical variables, in particular in terms of sex (*p* = 0.52), age (*p* = 0.18), baseline LVEF (*p* = 0.75) or RV AFS (*p* = 0.63), ICD implantation rate (*p* = 0.12), and genetic variants (*p* > 0.9). Anti-DSG2 antibody positivity was not different by genetic status, i.e., by the presence of pathogenic/likely pathogenic variants in ARVC-related genes.

With respect to AHA positivity, 26 ARVC patients (38%) were AHA-positive. ARVC-AHA-positive patients were more likely to also be AIDA-positive than ARVC AHA-negative patients (23% vs. 2%, *p* < 0001), while no further significant difference was observed in the other clinical variables (Supplementary Table S5).

**Figure 4:**
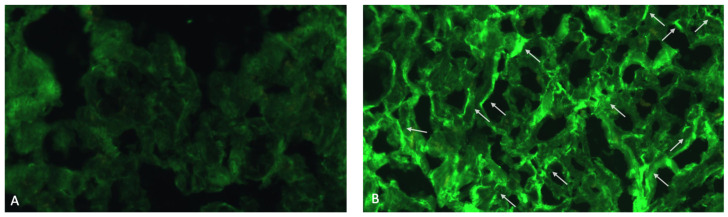
AIDA-positive pattern by Indirect Immunofluorescence (IFL). Panel (**A**) shows a negative control serum on human atrial tissue by IFL (×200). No staining is present on cardiomyocytes or intercalated disks. Panel (**B**) shows a positive linear AIDA staining pattern of the intercalated disks (arrows) on human atrial tissue by IFL (×200). No staining is present on cardiomyocytes.

Finally, with respect to AIDA positivity, 7 ARVC patients (10%) tested AIDA-positive (Table 5, Figure 4); AIDA-positive patients had a higher probability of being AHA-positive (*p* < 0.001). In addition, ARVC-AIDA-positive patients were more likely to present clinically with pre-syncope (60% vs. 12%, *p* = 0.025) and abnormalities in cardiac rhythm on baseline ECG (20% vs. 2%, *p* = 0.03) than ARVC-AIDA-negative patients.

## 4. Discussion

In our multicentric study, a relevant proportion (56%) of ARVC patients was anti-DSG2-ab-positive, with significantly higher titer than controls, confirming previous findings obtained in smaller cohorts [11]. For the first time, anti-DSG2-abs were also assessed in myocarditis/DCM patients, which tested positive in 48% of cases; moreover, the anti-DSG2-ab titer was similar between ARVC and myocarditis/DCM patients. The presence of autoantibodies towards desmosomal proteins in both ARVC and myocarditis/DCM highlights the shared immune-mediated background of these three conditions, in which the role of autoimmune-mediated mechanisms is established. For instance, anti-DSG2-abs have been found in other inflammatory cardiomyopathies, i.e., cardiac sarcoidosis, and showed a correlation with non-invasive imaging techniques demonstrating myocardial active inflammation [21].

Secondly, in the whole patients’ cohort, the frequency of anti-DSG2-ab-positive cases by ELISA by U/L was higher in AIDA-positive cases by IFL than AIDA-negative cases, in keeping with the hypothesis of DSG2 being one of the AIDA autoantigens [23]. In fact, AIDAs encompass a heterogeneous group of autoantibodies reacting with the intercalated disks, and DSG2, a cadherin calcium-dependent protein, is only one of the several desmosome structural proteins. Therefore, ELISA may be used as a screening method for anti-DSG2-abs in ARVC, to be confirmed by IFL, which remains the diagnostic gold standard. A correlation between the results of IFL and ELISA has been verified for other types of autoantibodies (i.e., antinuclear antibodies) in the setting of various immune-mediated diseases, but systematic data are lacking [24]; this may be due to the fact that different ELISA kits have different sensitivity and specificity for each specific antigen [25]. From a clinical perspective, in our study, anti-DSG2-ab status did not correlate with worse clinical and/or morphofunctional features in ARVC patients; on the other hand, in ARVC, it was AIDA positivity that led to a higher probability of being AHA-positive, presenting more pre-syncope and abnormalities in cardiac rhythm than ARVC AIDA-negative counterparts. This finding is possibly related to a wider AIDA autoantigenic spectrum and to the complex pathogenesis of ARVC. On one hand, it has been postulated that a mechanism for the presence of anti-DSG2-abs in ARVC is the exposition of DSG2 cryptic epitopes [26] after desmosome disruption; on the other hand, in ARVC, several mutations in different desmosomal proteins can be found, and in most cases the disease remains genetically elusive, i.e., without the possibility to identify a pathogenic mutation at genetic screening. In fact, in our ARVC cohort, only 3/77 (4%) patients were carriers of a pathogenic DSG2 genetic variant, leading to the possibility that mutations in other proteins led to AIDA, but not anti-DSG2-ab generation. Secondly, incomplete penetrance and variable expressivity of genetic variants in ARVC is well known, even for individuals belonging to the same family [27]. Notwithstanding, the adverse prognostic role of AIDA in ARVC corroborates the findings from previous experiences [16]. Moreover, AIDA positivity was observed in other cardiac diseases, such as idiopathic recurrent pericarditis, with similar negative prognostic value [28].

The correlation between AHA and AIDA positivity that we have observed in this ARVC cohort has already been identified in other diseases [18,19]; indeed, autoimmune/immune-mediated cardiac and/or extracardiac diseases often present an overlap of autoreactive antibodies. For this specific case, both AIDA and AHA are organ-specific antibodies: the AHA autoantigens are the α and β myosin heavy chain cardiac isoforms, and the AIDA autoantigens are the cardiac intercalated disks [29]. The specific autoantigen spectrum of AIDA has not been fully elucidated, and it is expectable that DSG2 is only one of the possible AIDA targets, since a variety of proteins are involved in the complex desmosomal architecture. A shared autoimmune background towards different cardiac autoantigens may explain the overlap between ARVC and myocarditis/DCM pathogenesis and clinical presentations.

The identification of the immune-mediated pathogenesis of ARVC has relevant clinical implications, since this could justify a deeper characterization of ARVC patients to assess a potentially treatable mechanism for the disease progression. The clinical overlap between ARVC “hot phases” and clinically suspected, or biopsy-proven, infarct-like myocarditis is already established [30]. On the other hand, myocarditis can present with an isolated RV involvement [31]. An etiopathogenetic overlap may explain the complexity of phenotypes that ARVC could have, including the recently defined left-dominant and biventricular forms of the disease. By all means, the latest ESC classification of cardiomyopathies classifies such forms as “non-dilated left ventricular cardiomyopathies” (NDLVC) [32] by a phenotypical approach; the possibility of an inflammatory origin of the morphofunctional cardiac alterations (essentially LV and/or RV scar) can be postulated in each case, similarly to what is already established for autoimmune/immune-mediated DCM [33].

By considering the growing body of evidence supporting the use of tailored immunosuppressive therapy in biopsy-proven inflammatory cardiomyopathy [34,35], the confirmation of active myocarditis in the EMBs of ARVC patients could lead to the etiological treatment of young patients and to a change in the natural history of the disease. The exact role of pathogenic genetic variants as triggers of a pathological immune reaction, or as bystanders in case of incomplete penetrance, variable expressivity or negative genetic testing should be evaluated in future studies.

In terms of future perspectives, the assessment of anti-DSG2-ab in family members of ARVC patients and/or in carriers of genetic variants with negative phenotype could aid in determining the predictive role of such markers in the disease expressivity and progression. Likewise, further studies are needed to assess the role of immunosuppression in animal models of ARVC, as well as the contribution of innate and adaptive immunity in the onset and progression of the disease.

## 5. Study Limitations

In our study, the cut-off for anti-DSG2-ab positivity was assessed based upon an exclusively internal validation evaluation; further studies and external validation are needed to validate these reference values for anti-DSG-2 positivity in ARVC and other cardiac and extracardiac diseases cohorts. Secondly, the low rate of pathogenic genetic variants in our ARVC cohort did not allow for a genotype–phenotype correlation (i.e., mutated cardiac protein-specific autoantibodies); future studies on wider ARVC cohorts are needed for establishing a robust genotype–phenotype analysis. Thirdly, the lack of prognostic value of anti-DSG-2 antibodies in our ARVC patients may have been due to the relatively small sample size and rate of adverse events observed in our cohort; further studies are needed to elucidate the role of anti-DSG-2 antibodies in larger prospective ARVC cohorts. Finally, the potential cross-reactivity of anti-DSG2-abs by ELISA, which has been previously described in other settings and diseases [36], may have led to an overestimation of anti-DSG2-ab positivity in our cohort; the diagnostic yield of ELISA in this setting should be investigated in future studies.

## 6. Conclusions

In our multicentric study, a relevant proportion of ARVC patients tested positive for anti-DG2-abs. This suggests an underlying immune-mediated pathogenesis towards desmosomal proteins, resembling immune-mediated mechanisms already established in myocarditis and autoimmune/immune-mediated cardiomyopathies. Moreover, the frequency of anti-DSG2-ab positivity by ELISA by U/L was higher in AIDA-positive cases by IFL than AIDA-negative cases, in keeping with the hypothesis that DSG2 is one of the AIDA autoantigens. Nevertheless, in ARVC patients, AIDA status, but not anti-DSG2-ab status, showed distinct clinical correlates, possibly reflecting a wider AIDA autoantigenic spectrum. Further studies are warranted to provide external validation for anti-DSG2-ab assessment, explore a genotype–phenotype analysis, and to identify the full autoantigenic targets related to the AIDA IFL pattern observed in ARVC and myocarditis.

## Figures and Tables

**Table 1:** ARVC patients’ (N = 77) clinical and immunological characteristics, including echocardiographic, arrhythmic, and genetic features. Legend: AHA: anti-heart antibody, AIDA: anti-intercalated disk antibody, anti-DSG-ab: anti-Desmoglein antibody, ARVC: arrhythmogenic right ventricular cardiomyopathy, DSG2: Desmoglein 2, DSP: Desmoplakin, ICD: implantable cardiac defibrillator, IV: intravenous, LVEF: left ventricular ejection fraction, NSVT: non-sustained ventricular tachycardia, OD: optical density, NYHA: New York Heart Association functional class, PKP2: Plakophilin 2, RV AFS: right ventricular area fractional shortening, VE 24 h: ventricular ectopic beats on 24 h Holter ECG monitoring, VT: ventricular tachycardia.

Variable	N (%)/Median (IQR)
Male sex	39 (56%)
*NYHA class at diagnosis:*	
I	50 (71%)
II	10 (14%)
III	1 (1%)
IV	2 (3%)
Symptoms at diagnosis	
Dyspnea	6 (9%)
Chest pain	8 (4%)
Palpitations	29 (11%)
Syncope	8 (11%)
Pre-syncope	10 (38%)
Sinus rhythm at diagnosis	61 (87%)
Presence of symptoms at follow-up	28 (40%)
ICD implantation	8 (11%)
IV beta-blocker treatment	15 (21%)
NSVT	12 (17%)
Sustained VT	1 (2%)
Age, years, median (IQR)	44, 50 (27.75–54.00)
VEs 24 h, median (IQR)	641 (8–10,224)
%RV AFS, median (IQR)	48.5 (37.75–56.00)
%LVEF, median (IQR)	62.00 (56.00–66.00)
Anti-DSG-ab levels, OD, median (IQR)	277.05 (170.75–423.375)
Anti-DSG-ab levels, U/L, median (IQR)	5.39 (3.07–9.175)
Genetic mutation	15 (19%)
*Genetic mutations:*	
DSG2 variants	3 (4%)
DSP variants	3 (4%)
PKP2 variants	9 (12%)
*AHA-positive:*	26 (37%)
Low positive	14 (20%)
Positive	7 (10%)
Strong positive	5 (7%)
*AIDA-positive:*	7 (10%)
Low positive	3 (4%)
Positive	4 (6%)
Both AHA- and AIDA-positive	6 (9%)

**Table 2:** Overall cohort (N = 245) descriptive statistics, including sex, age, and autoantibody assessment. Legend: AHA: anti-heart antibody, AIDA: anti-intercalated disk antibody, anti-DSG2-ab: anti-Desmoglein-2 antibody, ARVC: arrhythmogenic right ventricular cardiomyopathy, DCM: dilated cardiomyopathy, OD: optical density.

Variable		Data Available in	N (%)
Male sex		231	120 (49%)
*Diagnosis:*			
ARVC		245	77 (31%)
Myocarditis/DCM		245	91 (37%)
Autoimmune extracardiac disease		245	27 (11%)
Controls		245	50 (20%)
*AHA-positive:*		244	99 (40%)
Low positive			36 (15%)
Positive			43 (18%)
Strong positive			20 (8%)
*AIDA-positive:*		244	64 (26%)
Low positive			29 (12%)
Positive			29 (12%)
Strong positive			6 (2%)
Both AHA- and AIDA-positive		244	48 (20%)
Either AHA- and/or AIDA-positive		244	115 (47%)
	**Data available in**	**Median**	**IQR**
Age, years	220	43	28–54
Anti-DSG-ab titer, OD	245	247.7	152.00–398.00
Anti-DSG-ab titer, U/L	245	4.78	2.65–10.05
** Diagnosis group **	**Anti-DSG-2-ab, OD** **Median, IQR**	** Anti-DSG-2-ab, U/L** **Median, IQR **	
Healthy controls (N = 50)	179 (149–296)	3.46 (1.96–5.30)	
ARVC (N = 70)	276 (172–420)	5.50 (3.40–9.40)	
Myocarditis/DCM (N = 91)	255 (150–455)	4.90 (2.28–13.1)	
Autoimmune dis (N = 27)	248 (135–351)	5.00 (1.80–11.1)	

**Table 3:** Comparison of anti-DSG2-ab titer between diagnostic groups (i.e., healthy controls, ARVC, myocarditis/DCM, and autoimmune extracardiac diseases), according to anti-DSG2 antibody ELISA assessment by optical density (OD). ^1^ Kruskall–Wallis test. ^2^ Bonferroni correction for multiple comparison. Legend: ARVC: arrhythmogenic right ventricular cardiomyopathy, DCM: dilated cardiomyopathy.

Pairwise Comparison According to Diagnosis Groups		
Compared Groups	*p*-Value ^1^	p_adj_ ^2^
Controls—ARVC	**0.007**	**0.042**
Controls—Myocarditis/DCM	**0.020**	0.119
Controls—Extracardiac autoimmune diseases	0.535	1.000
Extracardiac autoimmune diseases—Myocarditis/DCM	0.231	1.000
Extracardiac autoimmune diseases—ARVC	0.127	0.761
Myocarditis/DCM—ARVC	0.609	1.000

**Table 4:** Comparison of anti-DSG2-ab titer between diagnostic groups (i.e., healthy controls, ARVC, myocarditis/DCM, and autoimmune extracardiac diseases), according to anti-DSG2 antibody ELISA assessment by U/L. ^1^ Kruskall–Wallis test. ^2^ Bonferroni correction for multiple comparison. Legend: ARVC: arrhythmogenic right ventricular cardiomyopathy, DCM: dilated cardiomyopathy.

Pairwise Comparison According to Diagnosis Groups		
Compared Groups	*p*-Value ^1^	p_adj_ ^2^
Controls—ARVC	**0.003**	**0.019**
Controls—Myocarditis/DCM	**0.009**	0.056
Controls—Extracardiac autoimmune diseases	0.149	0.894
Extracardiac autoimmune diseases—Myocarditis/DCM	0.606	1.000
Extracardiac autoimmune diseases—ARVC	0.391	1.000
Myocarditis/DCM—ARVC	0.610	1.000

**Table 5:** Comparison of immunological and clinical characteristics between AIDA-negative vs. AIDA-positive ARVC patients. Data are reported as N, (%). ^1^ Chi-squared test or Fisher exact test. Legend: AHA: anti-heart antibody, AIDA: anti-intercalated disk antibody, ARVC: arrhythmogenic right ventricular cardiomyopathy, IV: intravenous, LVEF: left ventricular ejection fraction, NSVT: non-sustained ventricular tachycardia, NYHA: New York Heart Association functional class, RV AFS: right ventricular area fractional shortening, VE 24 h: ventricular ectopic beats on 24 h Holter ECG monitoring, VT: ventricular tachycardia.

Variable	AIDA Neg, N = 62	AIDA Pos, N = 7	*p*-Value ^1^	Data Available in
Male sex	35 (57%)	4 (57%)	0.53	69
AHA pos	20 (32%)	6 (86%)	**<0.001**	69
NYHA class at diagnosis			0.122	63
I	46 (79%)	4 (80%)		
II	10 (17%)	0 (0%)		
III	1 (2%)	0 (0%)		
IV	1 (2%)	1 (20%)		
Dyspnea at diagnosis	5 (9%)	1 (20%)	0.40	63
Chest pain at diagnosis	7 (20%)	1 (20%)	0.51	63
Palpitations at diagnosis	25 (43%)	4 (80%)	0.13	63
Syncope at diagnosis	6 (10%)	2 (40%)	0.18	63
Pre-syncope at diagnosis	7 (12%)	3 (60%)	**0.025**	63
Symptoms at follow-up	25 (42%)	2 (40%)	0.94	63
ICD implantation	6 (10%)	2 (40%)	0.18	63
Sinus rhythm at diagnosis	57 (98%)	4 (80%)	**0.03**	63
IV beta-blocker treatment	14 (24%)	1 (20%)	0.66	63
NSVT	12 (25%)	0 (0%)	0.43	50
Sustained VT	1 (2%)	0 (0%)	1.00	50
Genetic mutation	12 (33%)	1 (20%)	0.48	41
Age (years)	34	34	0.99	69
VEs 24 h, mean	462	9429	0.49	39
%RV AFS, mean	48	33	0.18	62
%LVEF, mean	60	51	0.11	63

## Data Availability

Data are contained within the article.

## Supplementary Materials

The following supporting information can be downloaded at: https://www.mdpi.com/article/10.3390/jcm13226736/s1, Figure S1: ROC (Receiving Operator Curve) curve for anti-DSG2-antibodies, expressed as OD, in the whole study cohort (N = 245). AIDA: anti-intercalated disk antibodies, anti-DSG2 ab: anti-Desmoglein-2 antibodies; Table S1: Genotypic characterization of ARVC patients (N = 77). P/LP = pathogenic/likely pathogenic variants; Table S2: Comparison of frequencies of AIDA positivity by anti-DSG2-ab positivity in the overall cohort (N = 245), according to different cut-offs. ^1^ Chi-square test or Fisher exact test; Table S3: Comparison of frequencies of AHA positivity by anti-DSG ab positivity in the overall cohort (N = 245), according to different cut-offs. ^1^ Chi-square test or Fisher exact test; Table S4: Comparison of immunological and clinical characteristics between anti-DSG2 negative vs anti-DSG2 positive ARVC patients (according to UNITS 2 SD cut-off). Data are reported as N, (%). ^1^ Chi-square test or Fisher exact test, ^2^ Mann-Whitney test, ^3^ T-student test; Table S5: Comparison of immunological and clinical characteristics between AHA negative vs AHA positive ARVC patients. Data are reported as N, (%). ^1^ Chi-square test or Fisher exact test, ^2^ Mann-Whitney test, ^3^ T-student test.

## Abbreviations and Acronyms

AHA	anti-heart antibodies
AIDA	anti-intercalated disk antibodies
ARVC	arrhythmogenic right ventricular cardiomyopathy
DCM	dilated cardiomyopathy
DSG2	Desmoglien-2
ELISA	Enzyme-Linked ImmunoSorbent Assay
EMB	endomyocardial biopsy
ICD	implantable cardiac defibrillator
LVEF	left ventricular ejection fraction
OD	optical density
RV AFS	right ventricular area fractional shortening
NSVT	non-sustained ventricular tachycardia
VT	ventricular tachycardia
