# Supplementary material for: Prevalence and Correlates of Anti-DSG2 Antibodies in Arrhythmogenic Right Ventricular Cardiomyopathy and Myocarditis: Immunological Insights from a Multicenter Study
Source: J Clin Med. 2024 Nov 8;13(22):6736. doi: 10.3390/jcm13226736 (PMC11594951; doi:10.3390/jcm13226736)
Supplement: Supplementary file 1 [file jcm-13-06736-s001.zip › jcm-3297792-supplementary.pdf]

SUPPLEMENTARY MATERIAL

Supplementary Figure S1. ROC (Receiving Operator Curve) curve for anti-DSG2-antibodies, expressed as OD, in the whole study cohort (N=245). AIDA: anti-intercalated disk antibodies, anti-DSG2 ab: anti-Desmoglein-2 antibodies.

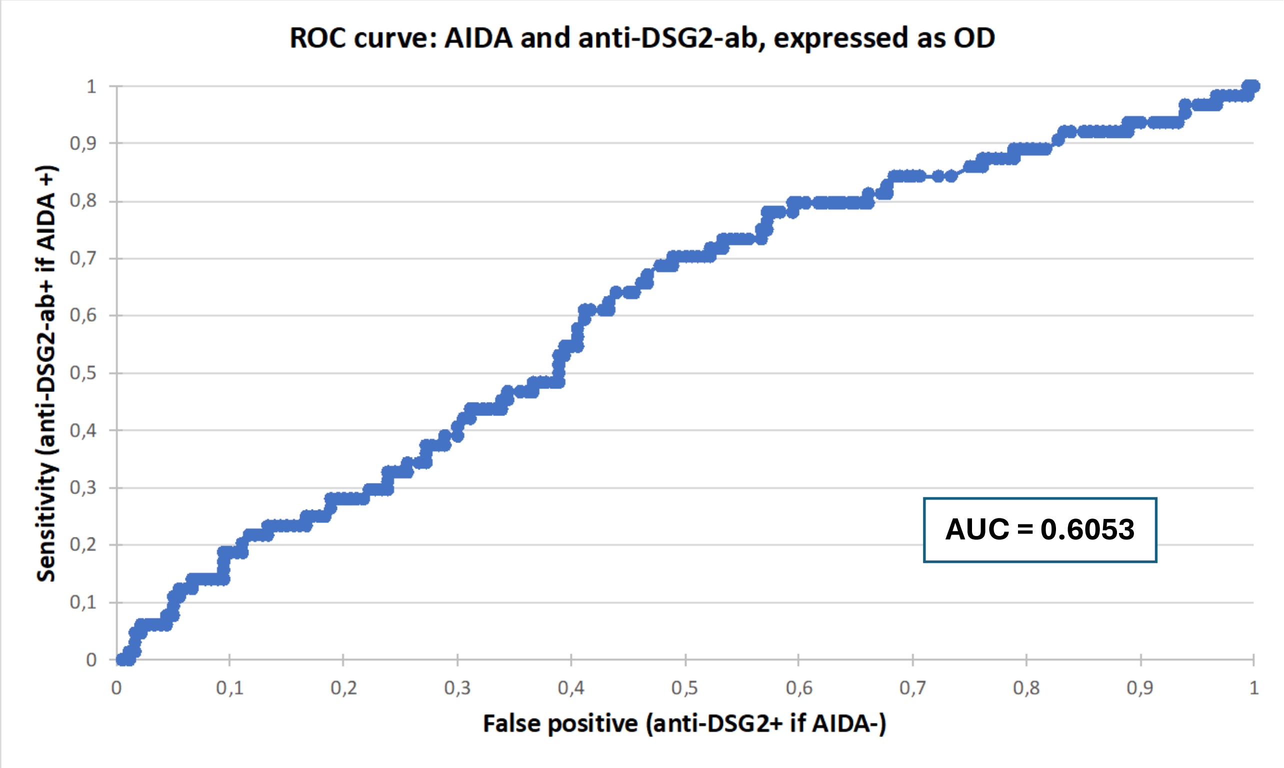

Supplementary Table S1. Genotypic characterization of ARVC patients (N=77). P/LP = pathogenic/likely pathogenic variants.

**Genetic test result**	**N (%)**
Negative	26/77 (34%)
PKP2 P/LP variants	3/77 (4%)
DSG2 P/LP variants	3/77 (4%)
DSP P/LP variants	9/77 (12%)
Genetic test not executed	36/77 (46%)

Supplementary Table S2. Comparison of frequencies of AIDA positivity by anti-DSG2-ab positivity in the overall cohort (N=245), according to different cut-offs. [1]Chi-square test or Fisher exact test.

Reference	Anti-DSG2-ab status	N	AIDA negative, N (%)	AIDA positive, N (%)	*p*-value[1]
Cut-off: U 2 SD	Anti-DSG2 ab negative	131	105 (80%)	26 (20%)	**0.033**
	Anti-DSG2 ab positive	113	75 (66%)	38 (34%)	
Cut-off: U 3 SD	Anti-DSG2 ab negative	148	118 (79%)	30 (20%)	**0.023**
	Anti-DSG2 ab positive	96	62 (64%)	34 (35%)	

Supplementary Table S3. Comparison of frequencies of AHA positivity by anti-DSG ab positivity in the overall cohort (N=245), according to different cut-offs. [1]Chi-square test or Fisher exact test.

		N	AHA negative, N (%)	AHA positive, N (%)	*p*-value[1]
Cut-off: OD 2 SD	Anti-DSG2 ab negative	118	78 (66%)	39 (33%)	0.051
	Anti-DSG2 ab positive	127	67 (53%)	60 (47%)	
Cut-off: U 2 SD	Anti-DSG2 ab negative	132	91 (69%)	40 (30%)	**0.002**
	Anti-DSG2 ab positive	113	54 (48%)	59 (52%)	
Cut-off: OD 3 SD	Anti-DSG2 ab negative	135	87 (64%)	47 (35%)	0.103
	Anti-DSG2 ab positive	110	58 (53%)	52 (47%)	
Cut-off: U 3 SD	Anti-DSG2 ab negative	148	101 (68%)	47 (32%)	**0.002**

Supplementary Table S4. Comparison of immunological and clinical characteristics between anti-DSG2 negative vs anti-DSG2 positive ARVC patients (according to UNITS 2 SD cut-off). Data are reported as N, (%). [1]Chi-square test or Fisher exact test, [2]Mann-Whitney test, [3]T-student test.

Legend: AHA: anti-heart antibodies, AIDA: anti-intercalated disk antibodies, anti-DSG2 ab: anti-Desmoglein-2 antibodies, ARVC: arrhythmogenic right ventricular cardiomyopathy, IV: intravenous, LVEF: left ventricular ejection fraction, NSVT: non-sustained ventricular tachycardia, OD: optical density, NYHA: New York Heart Association functional class, RV AFS: right ventricular area fractional shortening, SD: standard deviation, VE 24h: ventricular ectopic beats on 24hour-Holter ECG monitoring, VT: ventricular tachycardia.

Variable	Anti-DSG neg, N = 32	Anti-DSG pos, N = 38	*p*-value[1]	Data available in
Male sex	17 (53%)	15 (39.5%)	0.517	70
AHA pos	11 (34%)	2 (25%)	0.68	70
AIDA pos	2 (6%)	5 (13%)	0.36	70
Both AHA and AIDA pos	2 (6%)	4 (11%)	0.458	70
Either AHA and/or AIDA pos	11 (34%)	16 (42%)	0.468	70
NYHA class at diagnosis			0.143	63
• I	25 (93%)	25 (69%)		
• II	2 (7%)	8 (22%)		
• III	0 (0%)	1 (3%)		
• IV	0 (0%)	2 (6%)		
Dyspnea at diagnosis	1 (4%)	5 (14%)	0.226	63
Chest pain at diagnosis	3 (11%)	5 (14%)	1.00	63
Palpitations at diagnosis	13 (48%)	16 (44%)	0.485	63
Syncope at diagnosis	3 (11%)	11 (14%)	1.00	63
Pre-syncope at diagnosis	5 (19%)	5 (14%)	0.733	63
Symptoms at follow-up	13 (48%)	14 (39%)	0.348	63
ICD implantation	1 (4%)	7 (19%)	0.123	63
Sinus rhythm at diagnosis	27 (100%)	34 (94%)	0.461	63
IV beta-blocker treatment	7 (26%)	8 (22%)	0.772	63

NSVT	7 (32%)	5 (18%)	0.34	50
Sustained VT	0 (0%)	1 (4%)	1.00	50
Genetic mutation	5 (31%)	8 (32%)	1.00	41
Age (years)	31.5	36.5	0.168[2]	69
VEs 24h, mean	7278	9806	0.75[2]	39
%RV AFS, mean	48	46	0.63[3]	62
%LVEF, mean	61	59	0.75[3]	63

Supplementary Table S5. Comparison of immunological and clinical characteristics between AHA negative vs AHA positive ARVC patients. Data are reported as N, (%). [1]Chi-square test or Fisher exact test, [2]Mann-Whitney test, [3]T-student test.

Variable	AHA neg, N = 43	AHA pos, N = 26	*p*-value[1]	p_{adj}[2]	Data available in
Male sex	23 (54%)	16 (62%)	0.43	1.000	69
AIDA pos	1 (2%)	6 (23%)	**<0.001**	**0.019**	69
NYHA class at diagnosis			0.122	1.000	63
• I	35 (85%)	15 (68%)			
• II	5 (12%)	5 (23%)			
• III	0 (0%)	1 (5%)			
• IV	1 (2%)	1 (5%)			
Dyspnea at diagnosis	2 (5%)	4 (18%)	0.17	1.000	63
Chest pain at diagnosis	2 (5%)	6 (27%)	0.18	1.000	63
Palpitations at diagnosis	16 (39%)	13 (59%)	0.10	1.000	63
Syncope at diagnosis	5 (12%)	3 (14%)	0.58	1.000	63
Pre-syncope at diagnosis	5 (12%)	5 (23%)	0.29	1.000	63
Symptoms at follow-up	14 (34%)	13 (59%)	0.94	1.000	63
ICD implantation	4 (10%)	4 (018)	0.28	1.000	63
Sinus rhythm at diagnosis	40 (98%)	21 (96%)	0.30	1.000	63
IV beta-blocker treatment	10 (24%)	5 (23%)	0.57	1.000	63
NSVT	8 (26%)	4 (21%)	0.49	1.000	50
Sustained VT	1 (3%)	0 (0%)	0.62	1.000	50
Genetic mutation	6 (24%)	7 (44%)	0.16	1.000	41
Age (years)	32	37	0.19[2]	1.000	69

VEs 24h, mean	9999	5849	0.56[2]	1.000	39[1]
%RV AFS, mean	48	45	0.34[3]	1.000	62[2]
%LVEF, mean (SD)[3]	60	60	0.97[3]	1.000	63[2]
